# Simulation and Experiment of Active Vibration Control Based on Flexible Piezoelectric MFC Composed of PZT and PI Layer

**DOI:** 10.3390/polym15081819

**Published:** 2023-04-07

**Authors:** Chong Li, Liang Shen, Jiang Shao, Jiwen Fang

**Affiliations:** School of Mechanical Engineering, Jiangsu University of Science and Technology, Zhenjiang 212100, China

**Keywords:** piezoelectric polymer, flexible beam, LQR, differential evolution algorithm, vibration control

## Abstract

In order to improve the vibration suppression effect of the flexible beam system, active control based on soft piezoelectric macro-fiber composites (MFCs) consisting of polyimide (PI) sheet and lead zirconate titanate (PZT) is used to reduce the vibration. The vibration control system is composed of a flexible beam, a sensing piezoelectric MFC plate, and an actuated piezoelectric MFC plate. The dynamic coupling model of the flexible beam system is established according to the theory of structural mechanics and the piezoelectric stress equation. A linear quadratic optimal controller (LQR) is designed based on the optimal control theory. An optimization method, designed based on a differential evolution algorithm, is utilized for the selection of weighted matrix Q. Additionally, according to theoretical research, an experimental platform is built, and vibration active control experiments are carried out on piezoelectric flexible beams under conditions of instantaneous disturbance and continuous disturbance. The results show that the vibration of flexible beams is effectively suppressed under different disturbances. The amplitudes of the piezoelectric flexible beams are reduced by 94.4% and 65.4% under the conditions of instantaneous and continuous disturbances with LQR control.

## 1. Introduction

Piezoelectric materials have been widely used in the vibration control of structures due to their characteristics of light weight, flexible size, fast response speed, and wide frequency response range [1,2,3]. By using the positive and inverse piezoelectric effects of piezoelectric materials, sensors and actuators can be made embedded into the structure’s surface in order to control the active vibration of the system.

The piezoelectric materials commonly used for vibration control include single-layer piezoelectric ceramics, multilayer piezoelectric ceramics, and piezoelectric fiber composite materials. Single-layer piezoelectric ceramics are ferroelectric ceramics which possess a piezoelectric effect after being polarized by a high-voltage DC electric field. They have the advantages of low density, good response accuracy, high-frequency response, and high output [4,5,6]. Multilayer piezoelectric ceramics can generate more significant displacement than single-layer piezoelectric ceramics under the existing driving conditions in order to meet the need for vibration reductions in large vibration amplitude [7,8,9]. Although piezoelectric ceramics have achieved good results in vibration control, their application scope is limited due to low toughness and high brittleness. With the complexity and diversification of mechanical structures, flexibility has become one of the essential indicators of piezoelectric materials. Piezoelectric fiber composites can be bonded in various types of systems or embedded into composite structures, bending and twisting forms under the action of the applied voltage in order to generate or counteract vibration. They can be used as strain gauges to sense deformation, noise, and vibration when there is no applied voltage [10,11,12]. Piezoelectric fiber composites can also be used in precision sensor technology in microscopic mass sensing and measurements of viscosity, stiffness, and many other physical quantities using methods of influencing the electrical substitution model of piezoresonators [13,14].

In the field of active control with piezoelectric materials, researchers have carried out a series of studies and achieved numerous achievements. Hosseini et al. [15] proposed an active vibration control system for monitoring and suppressing human forearm fibrillation. It operates in the ways that are outlined here. Firstly, a dynamic model of the forearm is established. The forearm is simplified into a uniform flexible continuous beam model. The upper surface of the beam is covered with a layer of piezoelectric sensors, and the lower surface is covered with a layer of piezoelectric actuators, forming a vibration control system. The closed-loop active control of forearm vibration is realized by this control system. In addition, the effects of control gain, piezoelectric coefficient, and dielectric constant on the vibration response are investigated. The experimental results showed that the proposed active vibration control system can effectively suppress forearm flutter. Huang et al. [16] studied the active vibration control of piezoelectric sandwich plates. The structure used consists of two layers of piezoelectric sensors and a plate. The method used is based on the constitutive equation of piezoelectric material, whereby the active vibration control dynamic equation of the sandwich structure is established using the hypothesis mode method and Hamiltonian principle. The results show that the natural frequency of the structure is greatly affected by the different boundary conditions and the position of the piezoelectric plate. It can be said that the effect of active control is proportional to the velocity feedback coefficient. Additionally, Sohn et al. [17] used the piezoelectric actuators to control the active vibration of the ship shell structure. Watanabe et al. [18] analyzed and tested the active vibration control of the piezoelectric plate on the swing of the rear edge of the aircraft wing. National Aeronautics and Space Administration (NASA) researchers applied piezoelectric active control to the vibration of the rotating blade [19]. Furthermore, Callipari et al. [20] studied the active control of elastic vibration of large space structures by using bias piezoelectric actuators and tested the control model using the cantilever plate of solar panels. Hashemi et al. [21] designed an intelligent active vibration control system composed of a piezoelectric actuator and linear quadratic regulator in order to control the transverse deflection of wind turbine blades. To apply the control rules to fan blades, an advanced semi-analytical solution is proposed for the transverse displacement of fan blades under external loads.

Although piezoelectric ceramics have been widely used in piezoelectric vibration control, they can be used for the vibration reduction of some specific structures because of their poor elasticity. However, piezoelectric fiber composites have good elasticity and flexibility and they can adapt to different structural surfaces. This means that they have more applications in the field of vibration control. A novel composite sandwich beam with an adaptive active control system was proposed by Lu et al. [22]. The macro-fiber composite (MFC) piezoelectric patches were employed to construct the adaptive closed-loop control system. The resulted show that the vibrations induced by complex multi-frequency excitation with different amplitudes were effectively reduced with MFC piezoelectric patches. Wang et al. [23] investigated vibration suppression for a high-speed macro–micro manipulator with structural flexibility and parameter perturbation. In the study, the macro–micro manipulator contained an air-floating macro-motion platform and an MFC micromanipulator. The electromechanical dynamic model was thus derived. The results showed the proposed control strategy improved manipulation stability, robustness, and accuracy. Furthemore, the topology optimization of piezoelectric macro-fiber composite patches on laminated plates for vibration suppression was analyzed by Padoin et al. [24]. The linear–quadratic regulator (LQR) optimal control technique was used to find the optimum localization of the MFC piezoelectric patch for vibration control. The proposed MFC structure interaction model agreed well with experiments and numerical simulations of models. Dubey et al. [25] studied the shear-based attenuation of the vibration of an annular sandwich plate using shear actuated fiber composite (SAFC) and balanced laminate of PFC (BL-PFC) methods. The results showed that the BL-PFC is the best for shear-based attenuation of vibration of the annular sandwich plate.

For piezoelectric-based flexible beam systems, many scholars have performed numerous studies and obtained valuable results. Mohammed et al. [26] investigated the active vibration control of a cantilever beam. The optimal LQR controller was designed to reduce the vibration of the smart beam. The effect of piezoelectric vibration suppression was studied by changing the beam length. The results indicated that the maximum reduction percentage for settling time related to the free vibration of the smart beam reaches 80%. Lu et al. [27] studied the active vibration control of thin-plate structures with partial smart constrained layer damping (SCLD) treatment. In their research, the emphasis was placed on the feedback control system in order to attenuate the vibration of plates with SCLD treatments. When the external incentive was obtained from a single-frequency signal, vibration response amplitude attenuated by up to nearly 60%. Ezzraimi et al. [28] compared the control effects of different control algorithms and optimized the parameters of the controller by using genetic algorithms. The active vibration control using two types of LQR and PID controllers with different control parameters was tested and compared for the two recovery configurations of the piezoelectric elements. Furthermore, the active vibration control of composite cantilever beams was investigated by Huang et al. [29]. The linear quadratic regulator (LQR) feedback gain was optimized based on the particle swarm optimization (PSO) algorithm. The study showed that the optimal feedback gain of the controller effectively balanced the control effect and the control cost. The vibration of the cantilever was reduced by more than 50%. A control system was designed by Grzybek for the multi-input and simple-output piezoelectric beam actuators based on macro-fiber composite [30]. Additionally, the LQR control algorithm was used to generate control voltages. Furthermore, multiple PZT actuators were employed to suppress the vibration of the composite laminate plate by Her [31].

Piezoelectric polymers allow researchers to overcome the drawbacks of ceramic materials. The popular piezoelectric polymers can be divided into polyvinylidene fluoride (PVDF), polylactic acids (PLA), polyurethanes (PU) and PI [32,33,34,35]. Piezoelectric polymers have demonstrated better dielectric behavior and field strength, as well as an ability to tolerate high driving voltage [36,37]. It is because of the above characteristics that piezoelectric polymers can be used for the active vibration control of structures. At present, the efficiency of active control methods with piezoelectric polymers has been greatly improved. However, the issue of how to improve the vibration reduction effect of piezoelectric active control has been the focus of scholars’ research.

Therefore, the purpose of this paper is to improve the vibration reduction effect of the flexible beam based on soft piezoelectric film composed of PZT and PI Layer. To achieve the goal, a differential evolution algorithm is used to realize the optimal control effect of a vibration reduction on piezoelectric flexible beams. Compared with other algorithms, the differential evolution algorithm has the advantages of few setting parameters and high search efficiency; thus, it can complete the optimization of the weighted matrix in a short time. This paper will carry out research on the following aspects of this issue. First of all, a dynamic model of piezoelectric flexible beam technology is established. Then, a linear quadratic optimal controller (LQR) based on the differential evolution algorithm is designed. Finally, the effectiveness of active vibration control with piezoelectric polymers is verified by experimental tests.

## 2. Dynamic Model of Piezoelectric-Based Flexible Beam

**Figure 1:**
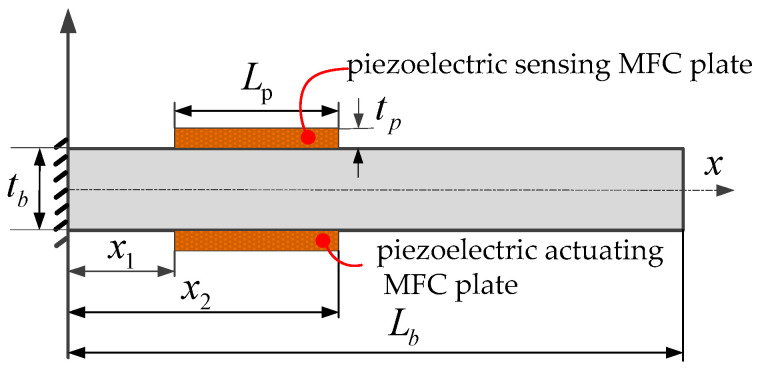
Piezoelectric flexible beam system.

A piezoelectric-based flexible cantilever beam structure is a simplified model commonly found in engineering mechanics. In this paper, the Euler–Bernoulli beam is selected as the research object. The piezoelectric flexible beam system consists of three parts: flexible beam, sensing piezoelectric MFC plate, and actuating piezoelectric MFC plate, as shown in Figure 1. 

The MFC-5628 was selected in this paper. It can be divided into P1 and P2 two types, and both of them can be used as piezoelectric actuators. The maximum displacement and output of P1 type are larger than that of P2 type. However, the driving voltage of P1 type ranges from −500 V to 1500 V, while that of P2 type only ranges from −60 V to 360 V. Therefore, under the same working conditions, the assessment of P2 type is simpler. Therefore, a P2 piezoelectric plate is selected in this paper. The piezoelectric MFC film is composed of a piezoelectric material that has two sides and one side is attached to an adhesive backing sheet. The slicing of the piezoelectric material constitutes a plurality of piezoelectric fibers in juxtaposition. A conductive film is then adhesively bonded to the other side of the piezoelectric material, and the adhesive backing sheet is removed. The conductive film has first and second conductive patterns formed thereon. These are electrically isolated from one another and are in electrical contact with the piezoelectric material. The first and second conductive patterns of the conductive film each have a plurality of electrodes with which to form a pattern of interdigitated electrodes. A second film is then bonded to the other side of the piezoelectric material. The second film may have a pair of conductive patterns similar to the conductive patterns of the first film [37,38].

The piezoelectric sensing and actuating MFC plates are pasted symmetrically into the upper and lower surfaces of the cantilever beam, and their parameter information is shown in Table 1. The length of the cantilever is *L_b_*, the width is *b*, and the thickness is *t_b_*. Furthermore, the length of the sensing/actuating piezoelectric plate is *L_p_*, the width is the same as that of the flexible beam, and the thickness is *t_p_*. The distance between the left and right ends of the beam’s fixed end is *x*_1_ and *x*_2_, respectively.

According to the theory of structural mechanics, when the flexible beam is subjected to external forces, its dynamic model is [40]

(1) $$E_{b}I_{b}\frac{\partial^{4}y(x,t)}{\partial x^{4}}+\rho_{b}A_{b}\frac{\partial^{2}y(x,t)}{\partial t^{2}}=\frac{\partial^{2}M(x,t)}{\partial x^{2}}$$

where *E_b_*, *I_b_*, *ρ_b_* and *A_b_* are the elastic modulus, neutral axis moment of inertia, density and cross-sectional area of the flexible beam, respectively, but in which *y*(*x*,*t*) is the deflection of the beam. *M*(*x*,*t*) is the moment that acts on the beam.

When the flexible beam vibrates, the amount of charge generated by the sensing piezoelectric sheet attached to the upper surface of the beam is *Q*(*x*,*t*). At this time, the voltage between the two surfaces of the piezoelectric plate is [41]

(2) $$U_{s}(x,t)=\frac{Q(x,t)}{C_{p}}=\frac{bt_{b}d_{31}E_{p}}{2C_{p}}\sum_{i=1}^{n}\left[\varphi_{i}{}^{\prime }(x_{2})-\varphi_{i}{}^{\prime }(x_{1})\right]q_{i}(t)$$

where *d*_31_ is the piezoelectric constant, *E_p_* is the elastic modulus of the piezoelectric plate, *Cp* is the capacitance of the piezoelectric plate, *φ_i_*(*x*) is the *i*-th order principal mode of the system, and *q_i_*(*t*) is the *i*-th order modal principal coordinate.

Letting

(3) $$C_{i}=K_{s}[\varphi_{i}^{\prime }(x_{2})-\varphi_{i}^{\prime }(x_{1})]$$

where *K_s_* = *bt_b_d*_31_*E_p_*/(2*C_p_*).

When driving voltage *U* is applied to the actuated piezoelectric plate, the piezoelectric plate will generate stress due to the inverse piezoelectric effect:

(4) $$\sigma_{1}=\frac{E_{p}d_{31}}{t_{p}}U$$

where *σ*_1_ is the stress generated by the piezoelectric plate.

The bending moment generated by the piezoelectric plate around the neutral axis of the flexible beam can be written as [42]

(5) $$M=\int_{\frac{t_{b}}{2}}^{\frac{t_{b}}{2}+t_{p}}\sigma_{1}bydy=K_{a}U\left[h(x-x_{1})-h(x-x_{2})\right]$$

where *h*(*x*) is the Heaviside function, *K_a_* is the electromechanical coupling coefficient, and *K_a_* = *bd*_31_*E_p_*(*t_b_ + t_p_*)/2.

Substituting Equation (5) into Equation (1), the vibration differential equation of the beam under the action of the actuated piezoelectric plate can be expressed as

(6) $$E_{b}I_{b}\frac{\partial^{4}y(x,t)}{\partial x^{4}}+\rho_{b}A_{b}\frac{\partial^{2}y(x,t)}{\partial t^{2}}=K_{a}U[\delta^{\prime }(x-x_{2})-\delta^{\prime }(x-x_{1})]$$

where δ′(*x* − *x_i_*) is the first order derivative of the Dirac function. The Dirac function can be expressed as

(7) $$\delta (x-x_{i})=\left\{\begin{array}{c} 0\;,\;(x\neq x_{i})\\ 1\;,\;(x=x_{i}) \end{array}\right.$$

According to the principle of modal superposition and considering the damping characteristics of the system, the vibration differential equation of the beam in modal coordinates can be written as

(8) $${\ddot{q}}_{i}(t)+2\xi_{i}\omega_{i}{\dot{q}}_{i}(t)+\omega_{i}^{2}q_{i}(t)=B_{i}U$$

where $B_{i}=K_{a}\left[\varphi_{i}{}^{\prime }(x_{2})-\varphi_{i}{}^{\prime }(x_{1})\right]$, *ξ_i_* is the *i*-th mode damping ratio of the system, and *ω_i_* is the natural frequency of the *i*-th mode of the system.

Introduce a state vector *x*(*t*)

(9) $$x(t)={\left\{q,\;\dot{q}\right\}}^{T}=\left\{q_{1}(t)\right.\;q_{2}(t)\;\cdots \;q_{n}(t),\;{\dot{q}}_{1}(t)\;{\dot{q}}_{2}(t)\;\cdots \;{\left.{\dot{q}}_{n}(t)\right\}}^{T}$$

Then, the state space equation of the piezoelectric flexible beam system can be expressed as

(10) $$\left\{\begin{array}{l} \dot{x}(t)=\left[\begin{array}{ll} 0_{n\times n} & I_{n\times n}\\ -\Omega & -2\Lambda \end{array}\right]x(t)+{\left[0_{1\times n}B_{1}\cdots B_{n}\right]}^{T}u(t)\\ y(t)=\left[C_{1}\cdots C_{n}0_{1\times n}\right]x(t) \end{array}\right.$$

where $\Omega =diag(\begin{array}{ccc} \omega_{1}{}^{2} & \cdots & \omega_{n}{}^{2} \end{array})$, $\Lambda =diag(\begin{array}{ccc} \xi_{1}\omega_{1} & \cdots & \xi_{n}\omega_{n} \end{array})$.

## 3. Piezoelectric Active Vibration Control System

### 3.1. LQR Controller Design

The controller is the core of the control system, and the controller’s performance will directly affect the performance and stability of the control system. In this paper, a linear quadratic optimal controller, which has clear performance indicators and simple control rules, is designed based on the differential evolution algorithm is designed. The differential evolution algorithm is used to optimize the weighted matrix *Q*. 

Define the system quadratic performance index function as

(11) $$J=\frac{1}{2}\int_{0}^{\infty }[x^{T}Qx+u^{T}Ru]dt$$

where the semi-positive definite matrix *Q* is the weighted matrix of the state variables, and the positive definite matrix *R* is the weighted matrix of the input variables.

The purpose of optimal control is to find an optimal input *u* so that the system quadratic performance index *J* is minimized.

The input law that determines the optimal control is

(12) $$u^{*}=-Kx$$

where *K* is the state feedback gain matrix.

(13) $$K=-R^{-1}B^{T}P$$

where *P* is a positive definite symmetric matrix, which is the solution of the Riccati algebraic equation:

(14) $$PA+\dot{P}+A^{T}P-PBR^{-1}B^{T}P+Q=0$$

Then, the optimal control law can be expressed as

(15) $$u^{*}=-Kx=-R^{-1}B^{T}Px$$

### 3.2. Selection of Weighted Matrices Q and R

In the quadratic performance index function *J*, the *x^T^Qx* term in the integrand mainly reflects the constraints or requirements of the state quantity during the system response, and the *u^T^Ru* term mainly reflects the constraints or requirements put on the control input during the system response. Therefore, the quadratic performance index function is essentially a balance between the amount of state change in the system and the energy consumed. The weighted matrices *Q* and *R* represent the penalties for the state quantity and control input, respectively. Decreasing the matrix *Q* is equivalent to increasing the matrix *R*.

In LQR control, the weighting matrices *Q* and *R* represent the penalties for state quantity and control input, respectively. Their penalties are relative, i.e., increasing the matrix *Q* is equivalent to decreasing the matrix *R*. In this paper, the weighted matrix *Q* is optimized by differential evolution algorithm. When the matrix *R* is enlarged or reduced by different multiples on the basis of the identity matrix *I*, the element values in the matrix *Q* will be equally enlarged or reduced by the same multiples. However, the optimal control feedback gain matrix *K* will not be changed, nor will the vibration control effect be changed. Therefore, if *R* = *I* is set, the elements in *Q* matrix are 10^7^ orders of magnitude, and the *J_c_* of optimization result is 10^4^ orders of magnitude, which indicates that the numerical series is too large. Therefore, if *R* = 0.01 *I* is set, the elements in the matrix *Q* are reduced to 10^5^ orders of magnitude. *J_c_* is reduced to 10^2^ orders of magnitude in the optimization result. At the same time, when *R* = 0.01 *I*, the control effect will not be affected.

To simplify the calculation, set the weighted matrix *R* to 0.01 *I* and the weighted matrix *Q* to be diagonal. Allow the weighted matrix *Q* to follow the form

(16) $$Q=diag[\begin{array}{cccc} q(1) & q(2) & \cdot \cdot \cdot & q(2n) \end{array}]$$

According to the characteristics of the piezoelectric flexible beam system, the optimization objective function of the system is defined as:

(17) $$J_{c}=\frac{1}{2}\int_{0}^{\mathrm{ts}}[x^{T}Qx+u^{T}Ru]dt$$

where *ts* is the set time, which is determined according to the actual operating condition of the system. 

In addition to considering the vibration suppression effect of flexible beams, the limitations of the system hardware should also be considered. Therefore, the following two constraints are proposed as

(18) $$\left|\frac{y(ts)}{y(0)}\right|\leq \sigma \%$$

(19) $$\left|u\right|\leq U_{\max}$$

where Equation (18) mainly considers the vibration suppression effect of the flexible beam, i.e., when the system applies the feedback control, the amplitude of the flexible beam should be less than *σ*% of the initial amplitude at *t_s_* moment. Here, *y*(*ts*) corresponds to the upper envelope curve formed by the vibration peak. Equation (19) mainly considers the input voltage range limitation of the actuated piezoelectric plate.

### 3.3. The Differential Evolution Algorithm

The differential evolution algorithm has the advantages of simple principles, strong robustness, fast convergence speed, and high precision. Therefore, the differential evolution algorithm is used to optimize the weighted matrix *Q*.

The principle of the differential evolution algorithm is similar to that of the genetic algorithm, being mainly composed of three operations: mutation, crossover and selection.

(1) Mutation operation

Using the difference strategy, the vector difference of any two individuals in the parent population is scaled and summed with a third individual to produce a mutant individual, and the expression of this is as follows

(20) $$V_{i,G+1}=X_{r1,G}+F(X_{r2,G}-X_{r3,G})$$

where the subscript *G* is the current algebra of the population, *r*1, *r*2 and *r*3 are random and mutually different integers between [1, *NP*], *NP* is the population size and satisfies *NP* ≥ 4, *V_i_* is the *i*-th mutant individual in the population, and *F* is the mutation operator which usually takes the constant in the range [0, 2].

(2) Cross operation

The method of probabilistic selection is used to form new candidates from the original target individuals and mutated individuals.

(21) $$U_{ji,G+1}=\left\{\begin{array}{l} V_{ji,G+1},\;r\leq CR\;or\;j=i\;\\ X_{ji,G},\;r>CR\;and\;j\neq i \end{array}\right.;\;j=1,2,\cdots ,D$$

where *r* is the random number in [0, 1], *CR* is the cross operator, the value is *CR*∈[0, 1], *U_ji_*_,_*_G_*_+1_ is the individual generated after the crossover, *j* is the dimension position of the intersection operation within the individual, and *D* is the dimension within the individual.

(3) Select Actions

The greedy selection strategy is adopted to allow the target individual and the candidate compete to enable the selection of the individual with the better fitness function.

(22) $$X_{ji,G+1}=\left\{\begin{array}{l} U_{ji,G+1},\;f{(X}_{ji,G})<f(U_{ji,G+1})\\ X_{ji,G},f{(X}_{ji,G})\geq f(U_{ji,G+1}) \end{array}\right.$$

where *X_ji_*_,*G*+1_ is the new generation of individuals produced by the final selection, and *f* is the fitness function.

### 3.4. Fuzzy Controller

In order to compare the performance of the LQR controller when optimized by differential evolution algorithms, a fuzzy controller with dual input and single output is designed for comparison, and a fuzzy controller with dual input and single output is designed for the vibration active control of piezoelectric flexible beams. The basic working principle behind this is to convert the measured input into a fuzzy quantity that can be described through fuzzy control rules, perform fuzzy inference on a fuzzy output value, and then transform the fuzzy output value into an accurate value that can be used for actual control through defuzzification operation.

A common two-dimensional fuzzy logic controller is selected in this paper according to the vibration characteristics of the flexible beam. The first input is set as error *E*, which represents the difference between the end of the flexible beam and the beam balance position during the vibration process and further represents the amplitude of the beam end. The second input is set as the error change rate *EC*, which represents the velocity value of the flexible beam end in the process of vibration. The output *U* is set as the feedback control signal of the system.

In the process of input ambiguity, the discussion domain of input *E* and *EC* is set as [−3, 3], and that of output *U* is set as [−5, 5]. Input/output adopts a seven-level fuzzy set, including descriptors of NL (negative large), NM (negative medium), NS (negative small), O (zero error), PS (positive small), PM (positive medium), and PL (positive large). In order to facilitate membership calculation, triangular membership functions are selected for input E, EC and output U. Their membership function curves are shown in Figure S1.

The research object of this paper is the vibration control of the beam. When the error *E* is larger, the beam amplitude will be larger. In order to make the beam return to the equilibrium position as soon as possible, the control quantity *U* should be larger, and vice versa. The greater the error rate *EC*, the greater the beam velocity. Using the Mamdani fuzzy inference method, the 7 × 7 = 49 fuzzy rules described by the matrix table are designed, as shown in Table S1.

Finally, the fuzzy output value is transformed into an accurate value which can be used for actual control by using the center of gravity method.

## 4. Frequency Sweeping Experiment

Frequency sweeping is one of the commonly used methods for harmonic response analysis in structural dynamics. Compared with performing modal tests such as the hammering method, the frequency response characteristics of the structure can be obtained efficiently and accurately through the sweeping vibration test and the natural frequency of the structure can be found. The procedure behind a sweep frequency experiment is shown in Figure S2.

The frequency sweep experiment of piezoelectric flexible beam system is carried out in this paper. The basic principle behind doing so is that the sweeping signal generated by the signal generator is amplified by the piezoelectric driver and then acts on the driving piezoelectric plate. The vibration of each mode of the flexible beam system is excited so that the sensing piezoelectric plate can generate induction signals. After processing the induced signal, the vibration response of the flexible beam system under the swept signal can be obtained. Because the vibration response of the beam is at a maximum at the natural frequency, the natural frequency of the flexible beam can be obtained through performing the sweep frequency experiment.

**Figure 2:**
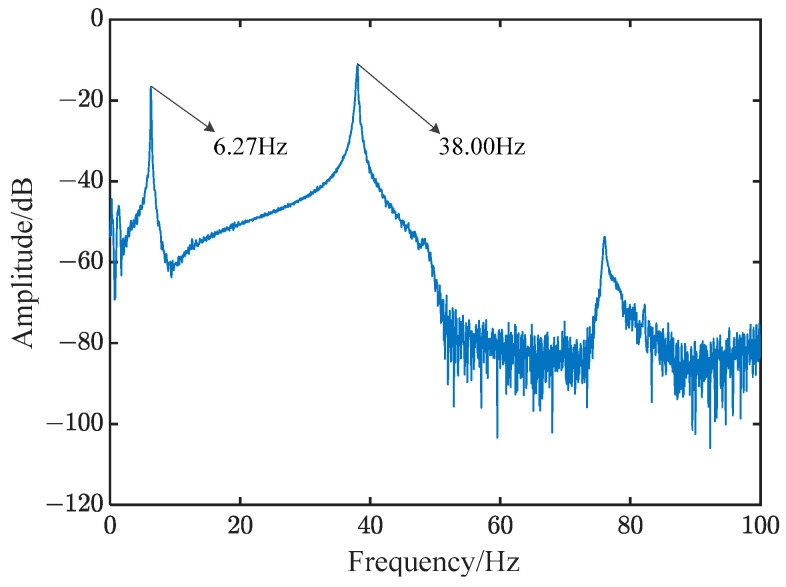
Results of frequency sweeping experiment.

Since the vibration energy of the flexible beam is mainly distributed in the lower-order mode, the frequency sweep experiment is only used for the first two order modes of the flexible beam. The correct parameters for its use are as follows: set the frequency range of the sweep to 0–50 Hz, the sweep mode to linear, the signal amplitude to 5 V, the sweep time to 15 s, and the sampling frequency to 500 Hz. After the experiment, the output signal of the sensing piezoelectric plate is shown in Figure S3, and the frequency response curve is shown in Figure 2.

The natural frequencies of the first two orders of the piezoelectric flexible beam system were obtained by performing an experiment and using the finite element method (FEM), as shown in Table 2. When simulated with FEM, the first two natural frequencies of the beam were 6.61 Hz and 39.04 Hz (see Figure S4). Additionally, the natural frequencies of the first two orders of the beam, obtained by using the frequency sweeping experiment, were 6.27 Hz and 38.00 Hz. It can be seen from Table 2 that the errors of the first two orders of natural frequency were 5.14% and 2.66%, respectively. The simulation results are consistent with the experimental results, which verify the correctness of the model.

## 5. Simulation

### 5.1. Optimal Positions of Sensing/Actuating Piezoelectric Plates

In order to optimize the position of the sensing/actuating piezoelectric MFC plates in the flexible beam, scholars have proposed a variety of optimization criteria from different perspectives. Examples include the controllability/observability criterion, system energy criterion, system stability criterion, etc.

Compared with other criteria, the D optimization criterion proposed by Bayard is simple and has clear physical meaning. Because the damping of the flexible beam structure is small, the D optimization method can be simplified. The optimal position of the sensing plate can be determined by the structural modal information of the beam. The sensing plate can be arranged in the place where the modal strain of the structure is at its maximum, and the actuating voltage plate can be symmetrically arranged on the upper and lower surfaces of the beam by centering.

According to the boundary conditions, the modal shape function and natural frequency of the flexible beam can be derived as follows:

(23) $$\varphi_{i}(x)=[\cosh\beta_{i}x-\cos\beta_{i}x+\gamma_{i}(\sinh\beta_{i}x-\sin\beta_{i}x)]\;\left(i=1,2,\cdot \cdot \cdot ,n\right)$$

(24) $$w_{i}={\left(\beta_{i}L_{b}\right)}^{2}\sqrt{\frac{E_{b}I}{\rho AL_{b}^{4}}}$$

where *β*_1_ = 1.875/*L_b_*, *β*_2_ = 4.694/*L_b_*, *γ_i_* = −(cos*β_i_L_b_* + cosh*β_i_L_b_*)/(sin*β_i_L_b_* + sinh*β_i_L_b_*).

The specific parameters of the flexible beam are shown in Table 3.

According to Equation (24), the first two orders of modal shape curves of the flexible beam can be obtained by MATLAB simulation, as shown in Figure S5.

The first two-stage open-loop vibration curves of the flexible beam are shown in Figure S6. It can be seen from the figure that the first-order open-loop vibration amplitude of the flexible beam undergoes slow attenuation and has a long vibration duration. The second-order open-loop vibration amplitude of the flexible beam decays quickly, and it can be seen that most of the vibration energy of the flexible beam is concentrated in the first-order mode. Therefore, in this paper, only the first-order modal vibration of flexible beams is controlled by vibration suppression.

The maximum strain of first-order modal vibration of flexible beams is located at the root of the beam. Therefore, according to the D optimization criterion, the piezoelectric actuator should be arranged at the root of the flexible beam and, according to the principle of symmetry, the sensing/actuated piezoelectric plate should be aligned and pasted to the root of the beam.

### 5.2. Vibration Control Simulation

**Figure 3:**
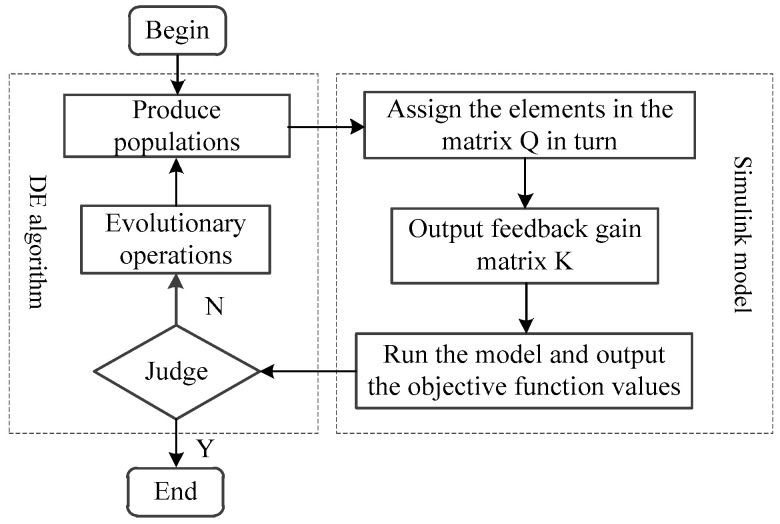
Algorithm optimization process.

The differential evolution algorithm is combined with the Simulink model of the piezoelectric flexible beam system to optimize the weighted matrix *Q*. Because the objective function *J_c_* is the minimum, the objective function *J_c_* is used as the fitness function *F*. The process of optimizing *Q* using the differential evolution algorithm is shown in Figure 3.

The specific steps in the differential evolution algorithm are as follows:

(1) The differential differentiation algorithm generates the initial population;
(2) The population individuals are assigned to each element in the weighted matrix *Q* in turn, and the optimal control feedback gain matrix *K* is calculated;
(3) The Simulink is run to model the flexible beam system, obtain the state quantity, control output voltage value, and calculate the objective function value;
(4) Researchers determine whether the termination conditions are met; if so, the optimization process is over, and the output is the result; otherwise, the population continues the evolutionary operation to obtain the evolved population, and researchers can skip to step 2.

In this paper, the first-order vibration model of the beam is selected as the research object. According to the established dynamic model, the state space equation of the piezoelectric flexible beam system can be obtained. In order to operate it, researchers should set the running time *t_s_* = 6 s, attenuation ratio σ % = 1%, and the maximum voltage *U_max_* = 360 V. The key parameter settings in the differential evolution algorithm are shown in Table 4.

The evolution of fitness function value during differential evolution is shown in Figure S7. As the process of differential evolution advances, the individuals with low fitness in the population are gradually eliminated, while the number of individuals with high fitness gradually increases. When this evolves to approximately 50 generations, the fitness function converges to the optimal value until the end of the entire optimization process. At this point, the objective function value becomes *J_c_* = 226.85, and the weighted matrix is *Q* = diag [6.19 × 10^5^ 3.83 × 10^5^].

In order to verify the performance of the LQR controller based on the differential evolution algorithm, a dual-input single-output (DISO) fuzzy logic controller is also designed. The peak value of load voltage calculated by the two control algorithms should be equal.

In Simulink, an active vibration control simulation platform for the piezoelectric flexible beam is built, and vibration control experiments under different disturbances are carried out.

The value of the weighted matrix *Q* of the common LQR controller is artificially obtained through the empirical method or trial and error method after substantial verification, a process which takes a long time and which has poor numerical accuracy.

**Figure 4:**
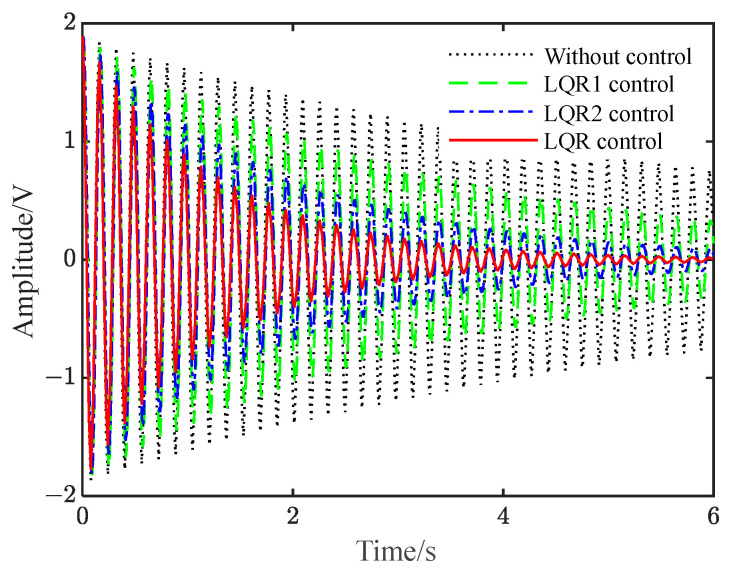
The vibration control effect varies with weighted matrix *Q*.

Figure 4 shows that the vibration control effect varies with weighted matrix *Q*, where the weighted matrix of LQR1 controller is artificially set as Q = diag [10^4^ 10^4^], and the weighted matrix of LQR2 controller is *Q* = diag [10^5^ 10^5^]. Therefore, the value of the weighted matrix *Q* needs to be adjusted artificially and continuously to induce the vibration control effect of the above constraints. The weighted matrix *Q* = diag [6.19 × 10^5^ 3.83 × 10^6^] of the LQR controller in the figure is obtained by searching the optimal solution value within the constraints using the differential evolution algorithm; this process only takes a short period of time and has high numerical accuracy. Obviously, since *Q* is optimal, the vibration suppression effect under the LQR control shown in Figure 4 is the best. When the LQR control parameter deviates from the optimal solution value, the control effect cannot be guaranteed, and the amplitude of the LQR control cannot be guaranteed to be smaller than that of fuzzy control. Thus, it is possible for the amplitude of the LQR control to be larger than that of fuzzy control. Therefore, in order to achieve the best control LQR effect, the optimal weighting matrix *Q* should be obtained.

LQR control is essentially a balance between the amount of system state change and the energy consumed, i.e., better control performance is achieved with less energy consumption. Ordinary LQR control weighted matrices can only be obtained empirically or via pro forma methods. Thus, “optimal” control is actually artificial. The purpose of using the differential evolution algorithm is to replace artificial labor and find the most ideal weighted matrix values in a short period of time. In order to compare the LQR performances optimized by the differential evolution algorithm, fuzzy control is used to compare the performance of the LQR controller under the same loading voltage. This proves that the optimization of the LQR controller using a differential evolution algorithm is successful.

**Figure 5:**
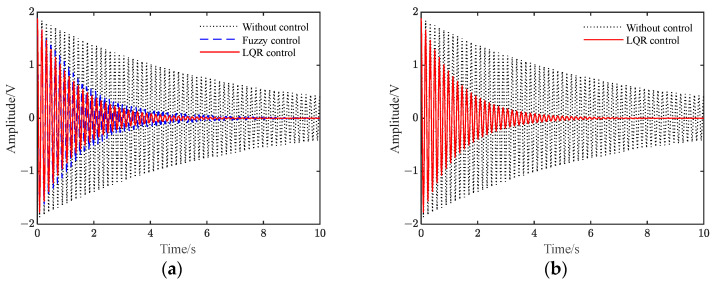
Vibration response under instantaneous disturbance. (**a**) Fuzzy and LQR control; (**b**) LQR control.

Figure 5 shows the vibration–response curve of the system under instantaneous disturbance. It can be seen from the figure that when there is no control, the vibration of the system decays slowly with time due to its damping. After applying LQR control and fuzzy control, the amplitude of the flexible beam is significantly suppressed over a short period of time, and the LQR control effect based on the differential evolution algorithm improves.

**Figure 6:**
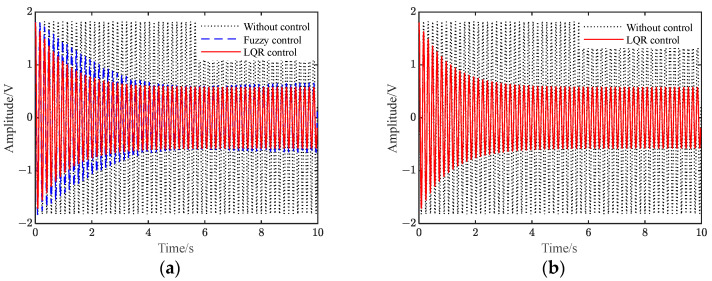
Vibration response under sinusoidal disturbance. (**a**) Fuzzy and LQR control; (**b**) LQR control.

The results that correspond to a sinusoidal signal *z* = 2sin(2πft) with a frequency of 6.27 Hz being applied as a disturbance signal are shown in Figure 6. It can be seen from the figure that when it is not controlled, the beam appears in the form of sinusoidal vibration. After the two controls are applied to the system, respectively, the vibration amplitude decreases first and then tends to become stable. Compared to the uncontrolled results, the amplitude is reduced by 68.7% and 63.8%. Additionally, the flexible beam under LQR control can reach a steady vibration suppression state faster. Compared to the fuzzy control, the LQR control is better.

**Figure 7:**
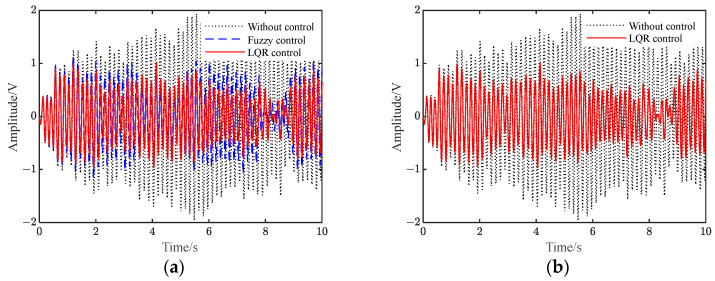
Vibration response under white noise disturbance. (**a**) Fuzzy and LQR control; (**b**) LQR control.

In addition, the response of the beam system under the white noise signal is studied, as shown in Figure 7. In Figure 7, a white noise signal with a PSD height of 100 and a sampling time of 0.01 s is investigated as the vibration response to the system disturbance signal. Results show when it is not controlled, the system undergoes irregular vibration, with a peak value of approximately 2 V. After applying the two controls separately, the vibration peak of the system is reduced to approximately 1 V, and the average vibration suppression effect under LQR control is better than that of fuzzy control.

## 6. Experiments and Results

### 6.1. Vibration Control Experiment under Instantaneous Disturbance

**Figure 8:**
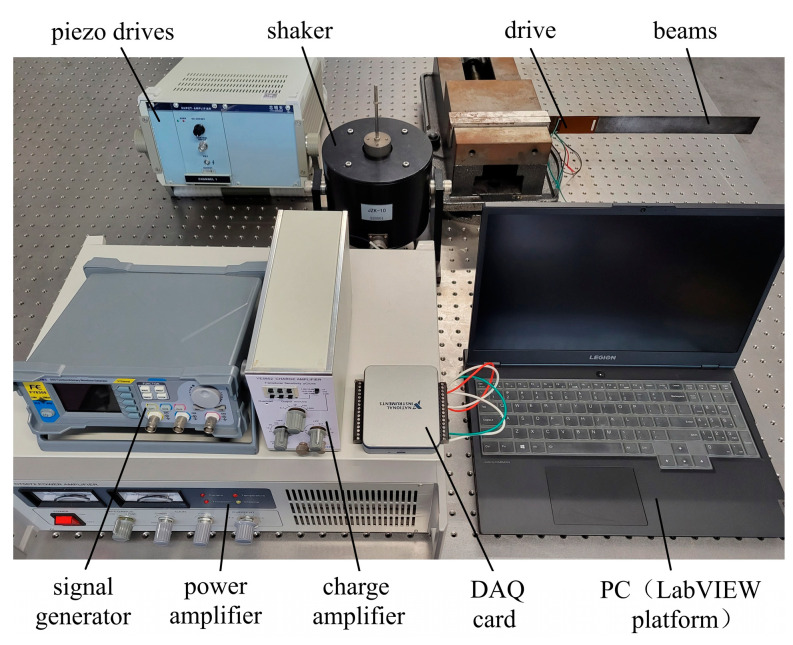
The experimental platform of the flexible beam system.

A structural diagram of the piezoelectric flexible beam active vibration control system is shown in Figure S8. Additionally, the system hardware experimental platform is shown in Figure 8. The system consists of a signal acquisition unit, an active control unit, a feedback actuation unit, and an excitation unit.

The signal acquisition unit comprises a sensing piezoelectric sheet MFC-5628 and a charge amplifier YE5852, the latter of which converts the vibration signal of the collected flexible beam into an equal proportion of the voltage signal. The active control unit is composed of a data acquisition card NI USB-6002 and a computer installed with LabVIEW. Its function is to calculate the corresponding control signal through the active control algorithm. The feedback actuation unit is composed of piezoelectric driver E01 and actuating piezoelectric sheet MFC-5628. Its function is to make the piezoelectric plate generate deformation under the action of the control voltage. Then, the suppression of beam vibration is realized. The excitation unit is composed of three parts: signal generator FY8300, power amplifier CT5872 and exciter JZK-10. These components are responsible for interfering with the flexible beam and then making the beam generate vibration.

In this experiment, an external force is applied to the end of the flexible beam to deviate it from the equilibrium position. Additionally, after the external force is removed, the flexible beam vibrates. The two designed controllers are used to perform vibration control experiments under the instantaneous disturbance of flexible beams.

**Figure 9:**
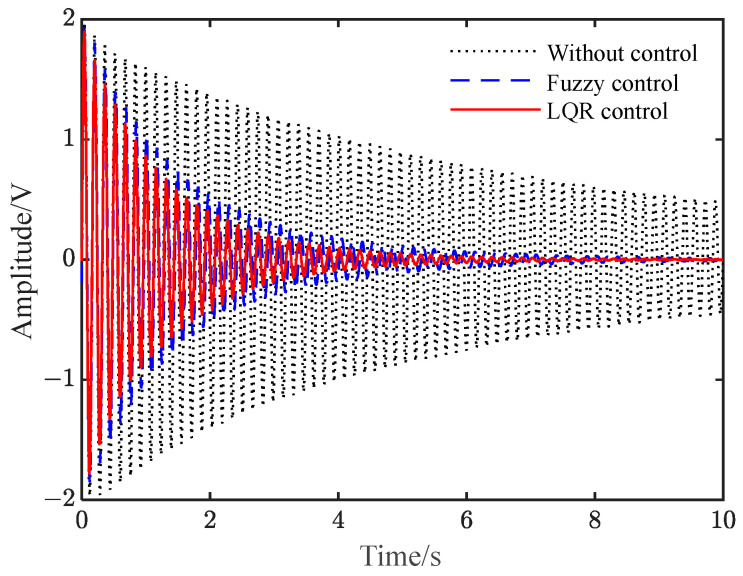
Vibration response under instantaneous disturbances.

Figure 9 shows the vibration response of the flexible beam under the conditions of instantaneous disturbance. Transient disturbance refers to the sudden change in the force acting on the flexible beam, which makes the whole piezoelectric flexible beam system appear transient response. When the flexible beam is at rest, it should be located at the equilibrium position, and its state should be zero. In the experiment, an external force is used to offset the end of the beam by a fixed distance from the equilibrium position. During the experiment, when the moment the external force is removed, the beam will vibrate. Before and after the experiment, the beam will have a transient response due to the change in the external force.

Figure S9 shows the voltage when applied to the piezoelectric plate under different controls. It can be seen from Figure 9 and Figure S9 that when there is no control, the vibration of the flexible beam is slowly attenuated. Additionally, after the two controls are applied, the amplitude of the flexible beam is quickly suppressed to a significant extent. Furthermore, the amplitude of the beam under LQR control is smaller than that under fuzzy control. 

Table 5 shows the amplitude comparison between the simulation and the experiment under instantaneous perturbation conditions. It can be seen from the table that after applying fuzzy control and LQR control, the beam amplitude drops to 0.10 V and 0.04 V in the simulation, which is a decrease of 88.6% and 95.5% compared with the uncontrolled 0.88 V. In the experiment, the beam amplitude decreases from an uncontrolled 0.90 V to 0.11 V and 0.05 V, a decrease of 87.8% and 94.4%, respectively. The simulation and experimental results are consistent.

### 6.2. Vibration Control Experiment under Continuous Disturbance

The excitation unit is used to apply a sinusoidal signal with a frequency of 6.27 Hz and amplitude of 2 V to the root of the flexible beam to excite the first-order modal vibration of the flexible beam. The two designed controllers are used to carry out vibration control experiments on flexible beams under continuous disturbance.

**Figure 10:**
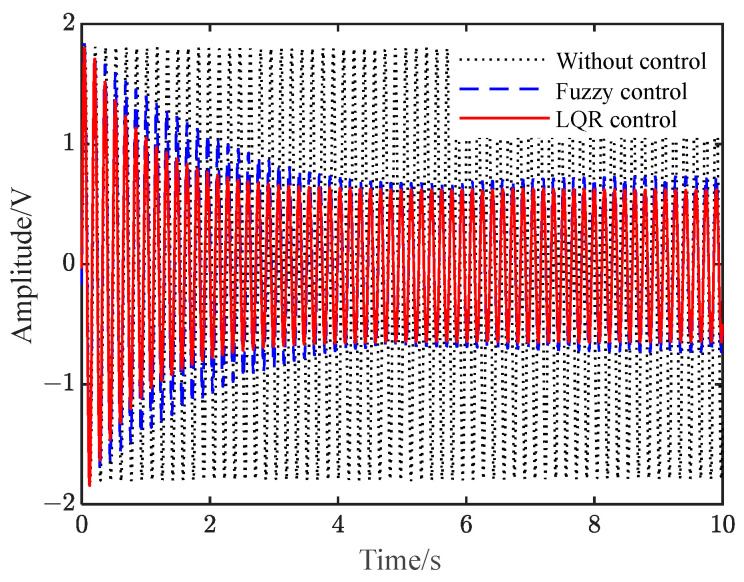
Vibration response under continuous disturbance.

Figure 10 shows the time-domain response under continuous disturbance. Figure S10 shows the voltage applied to the piezoelectric plate. It can be seen from the figure that when there is no control, the flexible beam displays sinusoidal vibration, with a vibration amplitude of 1.79 V. In our experiment, after the two controls were applied, the vibration amplitude of the flexible beam decreased to 0.62 V and 0.73 V, which was 65.4% and 59.2% lower than the uncontrolled amplitude, respectively. Additionally, the flexible beam under LQR control reached a stable vibration suppression state faster.

Table 6 shows the amplitude comparison of the simulation and experiment results under sinusoidal disturbance. It can be seen that after we had applied the LQR control and fuzzy control, the beam amplitude decreased from uncontrolled 1.82 V to 0.57 V and 0.66 V, which marked decreases of 68.7% and 63.8%, respectively. In the experiment, the beam amplitude decreased from an uncontrolled 1.79 V to 0.62 V and 0.73 V, which marked decreases of 65.4% and 59.2%, respectively. Hence, the simulation and experimental results were basically consistent. 

### 6.3. Vibration Control Experiment under White Noise Disturbance

The white noise signal with a PSD height of 100 and a sampling time of 0.01 s is applied to the root of the flexible beam. The vibration control experiment under the white noise disturbance is carried out on the flexible beam using the two controllers.

**Figure 11:**
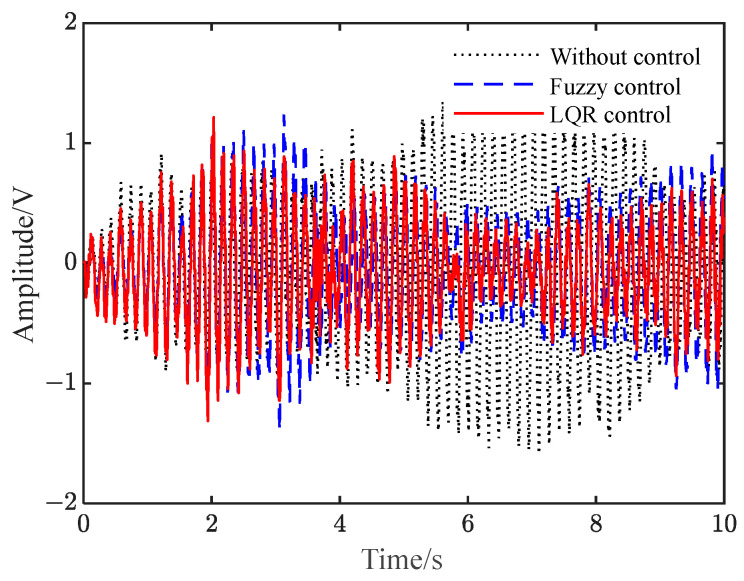
Vibration response under white noise disturbance.

Figure 11 shows the time domain response under white noise disturbance conditions. Figure S11 presents the voltage applied to the piezoelectric plate under white noise disturbance conditions. In general, the amplitude of the flexible beam is suppressed after the control is applied. Additionally, the suppression effect of the LQR control is slightly better than that of the fuzzy control. 

Table 7 shows the amplitude of frequency response under white noise disturbance. It can be seen that after the two controls were applied in our experiment, the peak value of the amplitude at the first-order resonance of the flexible beam was reduced from 0.74 V to 0.38 V and 0.58 V, respectively. Additionally, the vibration of the flexible beam was suppressed. Compared with the fuzzy control, the amplitude of the beam under LQR control was smaller, and the control effect was better. This was consistent with the simulation results, which verifies the correctness of the simulation results.

### 6.4. Innovation and Comparative Analysis

In this paper, the piezoelectric active vibration control of flexible beam is realized by using LQR controller based on difference algorithm. The innovation of this paper is mainly reflected in the following three aspects:

(1) The optimization of weighted matrix *Q* in LQR controller is traditionally obtained by using a genetic algorithm. However, genetic algorithms have the disadvantages of complex structure, amounts of setting parameters, and low search efficiency. However, the differential evolution algorithm has the advantages of simple principles, few setting parameters and high search efficiency. Compared with the genetic algorithm, the differential evolution algorithm can complete the optimization of the weighted matrix *Q* in a shorter time.

(2) As shown in Figure 10, when the weighted matrix *Q* reaches the optimal value, the average fitness of the LQR controller based on the difference algorithm becomes stable after 40 iterations. However, the LQR controller based on genetic algorithm achieved the stability of the average fitness after more than 60 iterations [43]. Furthermore, compared with the genetic algorithm, LQR controller based on differential evolution algorithm is more efficient.

(3) In the studies [20,21,30], maximum vibration attenuation amplitudes of 80%, 60% and 50% were achieved by piezoelectric active control in combination with other algorithms. In this paper, the differential evolution algorithm is used to achieve the maximum vibration attenuation amplitude of 94.4% of the flexible beam under instantaneous disturbance with LQR control. These results further show that the control effect obtained based on differential evolution algorithm is better.

## 7. Conclusions

In this paper, active control based on soft piezoelectric macro-fiber composite (MFC) consisting of polyimide (PI) sheet and lead zirconate titanate (PZT) is used to reduce the vibration. A dynamic coupling model of the piezoelectric flexible beam system is established. A linear quadratic optimal controller (LQR) is designed based on the optimal control theory. In order to verify the performance of the LQR controller optimized by the differential evolution algorithm, the fuzzy controller is used as a comparison, and simulation experiments are carried out in Simulink for different disturbance states. A systematic hardware experimental platform is established, and experimental research on active vibration control of flexible beams under different disturbances is carried out. The results show that:

(1) The design control algorithm has a good control effect on the vibration of flexible beams under different disturbance states. The vibration suppression effect of LQR control optimized based on the differential evolution algorithm is better than that of fuzzy control.

(2) The amplitudes of the piezoelectric flexible beams are reduced by 94.4% and 65.4% under instantaneous and continuous disturbances with LQR control.

(3) In the frequency sweeping experiment, the errors of the first two orders of natural frequencies are 5.14% and 2.77%, respectively.

## Figures and Tables

**Table 1:** Parameters information of piezoelectric MFC plate [39].

Parameter	Values	Parameter	Values
Working mode	*d*_31_	*d*_31_	−171 × 10^−12^ C/N
Thickness	300 μm	Effective working length	56 mm
Electrode	Standard lead-free solder S-Sn99Cu1	Effective working width	28 mm
Capacitance	187 nF	Total length	67 mm
Upper limit of operating frequency	<1 MHz	Total width	31 mm
Elastic compliance coefficient $s_{11}^{E}$	16.4 × 10^−12^ m^2^/N	Permittivity $\varepsilon_{33}^{T}$	−0.9

**Table 2:** The natural frequency of piezoelectric flexible beams.

	FEM Results	Experiment Results	Errors (%)
First order natural frequency/Hz	6.61	6.27	5.14%
Second order natural frequency/Hz	39.04	38.00	2.66%

**Table 3:** Flexible beam parameters.

Item	Flexible Beams
Length/width/thickness/mm	330/32/0.8
Density *ρ*/(kg/m^3^)	7850
Modulus of elasticity E/Gpa	198.6
Damping ratio *ξ*	0.004
1st round frequency/(rad/s)	38.9

**Table 4:** Key parameters of the differential evolution algorithm.

NP	F	CR	G
30	0.5	0.9	200

**Table 5:** Comparison of simulation and experimental results under instantaneous disturbance.

	Without Control	Fuzzy Control	LQR Control
Simulate amplitude (V)	0.88	0.10	0.04
Experimental amplitude (V)	0.90	0.11	0.05

**Table 6:** Comparison of simulation and experimental results under sinusoidal disturbance.

	Without Control	Fuzzy Control	LQR Control
Simulate amplitude (V)	1.82	0.66	0.57
Experimental amplitude (V)	1.79	0.73	0.62

**Table 7:** The amplitude of frequency response under white noise disturbance.

	Without Control	Fuzzy Control	LQR Control
Amplitude (V)	0.74	0.58	0.38

## Data Availability

Not applicable.

## Supplementary Materials

The following supporting information can be downloaded at: https://www.mdpi.com/article/10.3390/polym15081819/s1, Figure S1: Membership function graph of fuzzy controller; Table S1: Fuzzy control rule; Figure S2: The procedure of sweep frequency experiment; Figure S3: The output signal of the sensing piezoelectric plate; Figure S4: FEM analysis results of natural frequencies; Figure S5: Flexible beam modal curves; Figure S6: Open-loop vibration curves; Figure S7: Evolution of fitness function values; Figure S8: Schematic diagram of the experimental system; Figure S9: The voltage applied to the piezoelectric plate under instantaneous disturbance; Figure S10: The voltage applied to the piezoelectric plate under continuous disturbance; Figure S11: The voltage applied to the piezoelectric plate under white noise disturbance.
