# Supplementary material for: Simulation and Experiment of Active Vibration Control Based on Flexible Piezoelectric MFC Composed of PZT and PI Layer
Source: Polymers (Basel). 2023 Apr 7;15(8):1819. doi: 10.3390/polym15081819 (PMC10145578; doi:10.3390/polym15081819)
Supplement: Supplementary file 1 [file polymers-15-01819-s001.zip › polymers-2258783-supplementary.pdf]

Supplementary Materials

Simulation and Experiment of Active Vibration Control Based on Flexible Piezoelectric MFC Composed of PZT and PI Layer

Chong Li *, Liang Shen, Jiang Shao and Jiwen Fang

School of Mechanical Engineering, Jiangsu University of Science and Technology, Zhenjiang 212100, China
* Correspondence: lichong@just.edu.cn; Tel.: +86-511-8444-5385

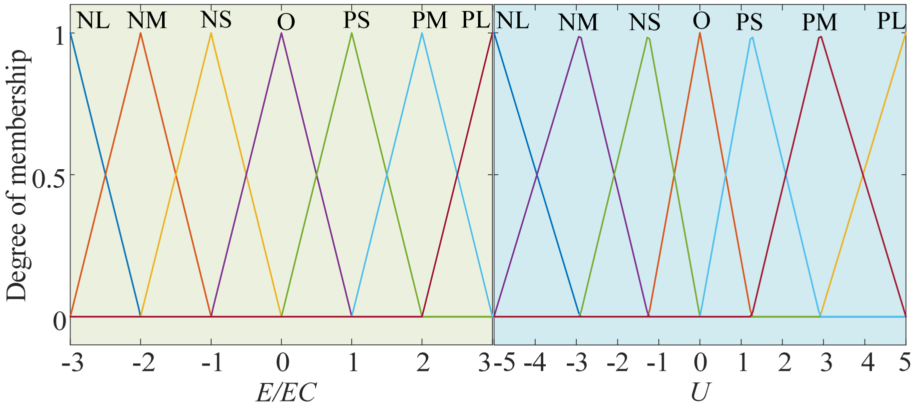

Figure S1. Membership function graph of fuzzy controller.

Table S1. Fuzzy control rule.

	U	*EC*						
		NL	NM	NS	O	PS	PM	PL
	NL	NL	NL	NL	NM	NM	NS	O
	NM	NL	NM	NM	NS	NS	O	PS
	NS	NL	NM	NS	NS	O	PS	PM
E	O	NM	NS	NS	O	PS	PS	PM
	PS	NM	NS	O	PS	PS	PM	PL
	PM	NS	O	PS	PS	PM	PM	PL
	PL	O	PS	PM	PM	PL	PL	PL

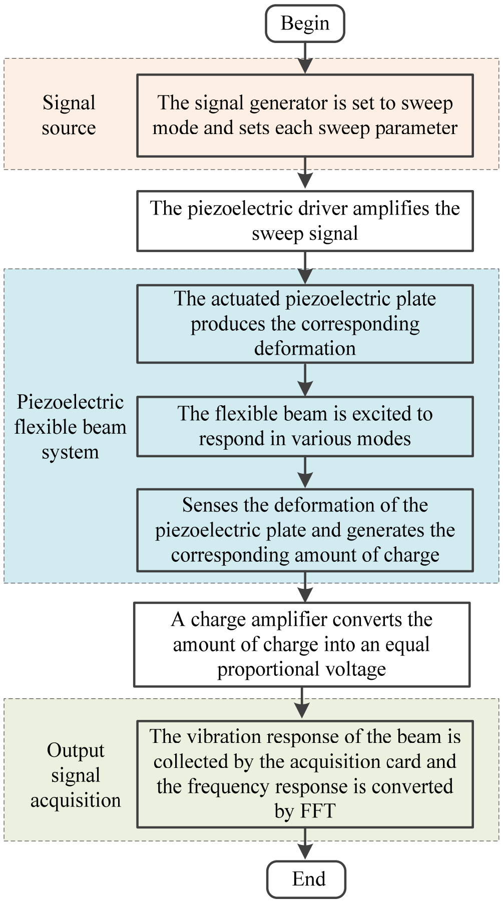

Figure S2. The procedure of sweep frequency experiment.

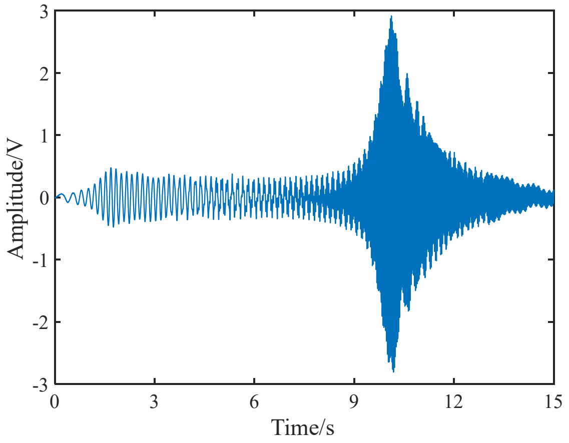

Figure S3. The output signal of the sensing piezoelectric plate

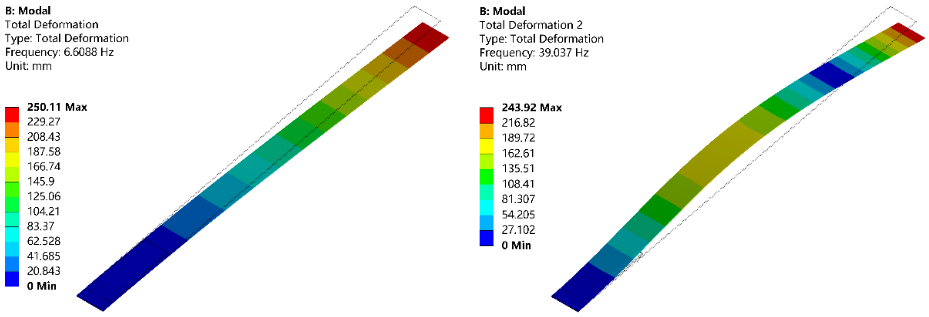

(a) (b)

Figure S4. FEM analysis results of natural frequencies. (a) First order frequency; (b) Second order frequency.

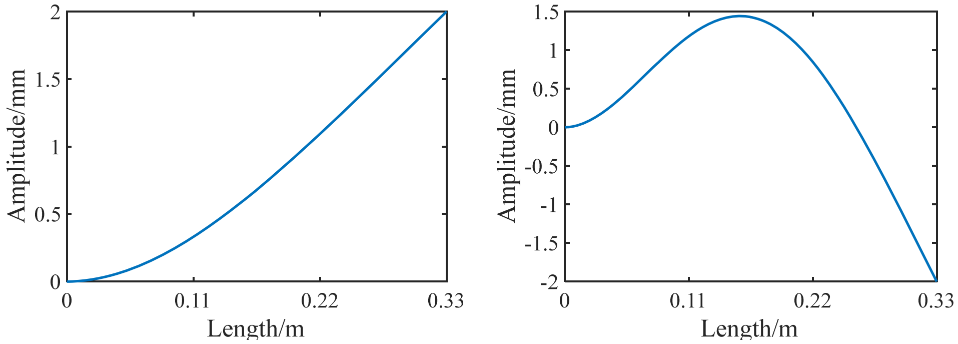

(a) (b)

Figure S5. Flexible beam modal curves. (a) First-order modal curve; (b) Second-order modal curve.

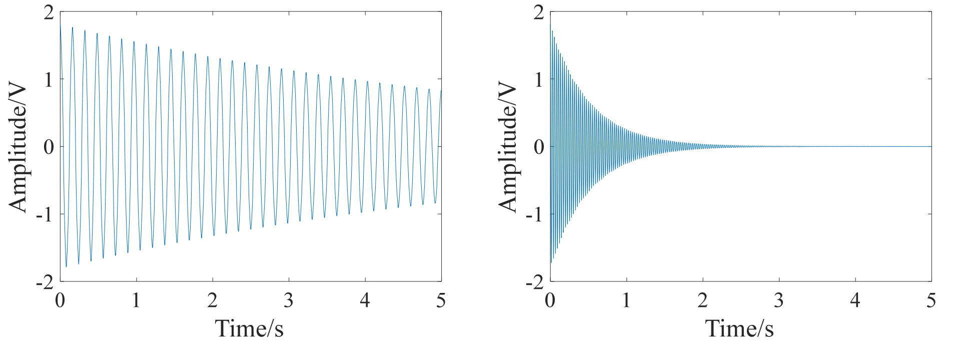

(a) (b)

Figure S6. Open-loop vibration curves. (a) First-order open-loop vibration curve; (b) Second-order open-loop vibration curve.

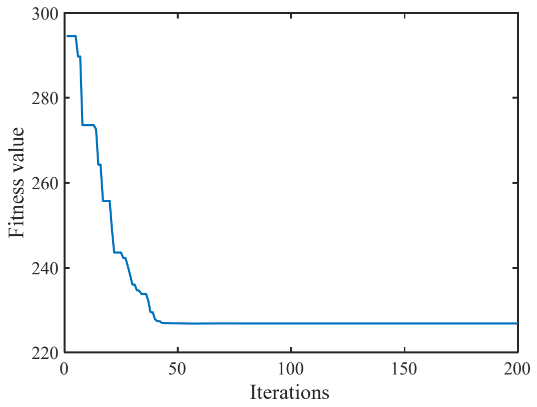

Figure S7. Evolution of fitness function values

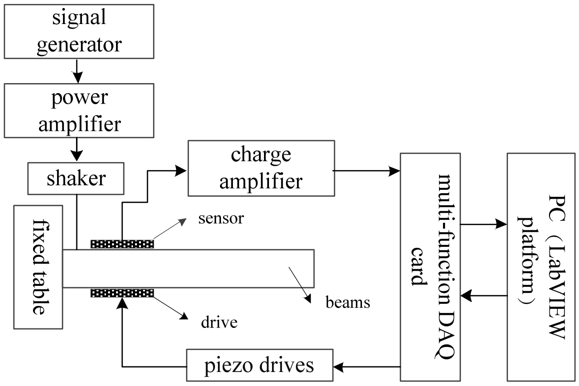

Figure S8. Schematic diagram of the experimental system

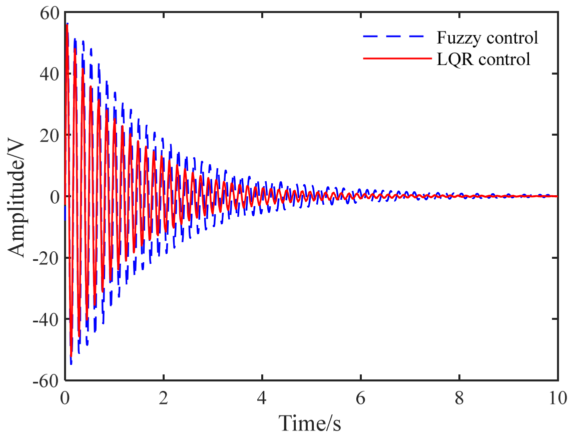

Figure S9. The voltage applied to the piezoelectric plate under instantaneous disturbance

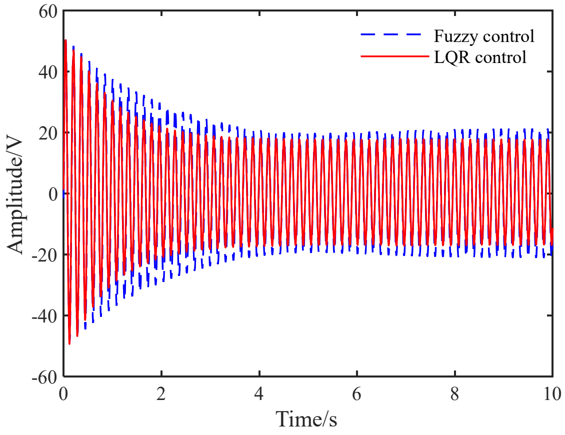

Figure S10. The voltage applied to the piezoelectric plate under continuous disturbance

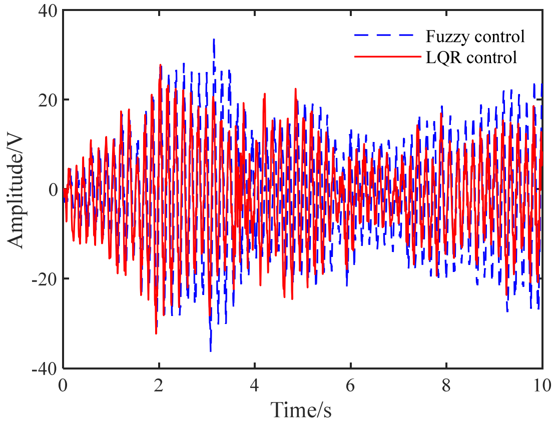

Figure S11. The voltage applied to the piezoelectric plate under white noise disturbance
